# Comprehensive bioinformatics analysis of the expression, prognostic value, and immune infiltration of chromobox family members in cervical cancer

**DOI:** 10.22038/IJBMS.2023.64845.14281

**Published:** 2023-04

**Authors:** Dan Liao, Xiaomei Liu, Limei He, Yuhong Yao, Xiuying Yuan, Poling Feng, Cuifen Li, Yanyan Liu

**Affiliations:** 1 Department of Gynaecology, SSL Central Hospital of Dongguan, Dongguan Third People’s Hospital, Affiliated Dongguan Shilong People’s; 2 Hospital of Southern Medical University, Dongguan, Guangdong, China; 3 Department of Gynaecology, Shixing Maternal and Child Health Hospital, Shaoguan, Guangdong, China

**Keywords:** Cervical cancer, Chromobox family, Expression, Immune infiltration, Prognosis

## Abstract

**Objective(s):**

Cervical cancer (CC) is the fourth most prevalent type of cancer in women worldwide and it is considered the leading cause of tumor-related death and malignancy. As part of complexes involved in epigenetic control, the proteins of the chromobox (CBX) family have been found to have a role in the growth of malignancies by preventing differentiation and increasing proliferation. Here, by a thorough investigation, we investigated the expression, prognostic significance, and immune infiltration of CBX in patients with CC.

**Materials and Methods:**

Differential expression, clinicopathological parameters, immune cell infiltration, enrichment analysis, genetic alteration, and prognostic value of CBXs in patients with CC were examined using TIMER, Metascape, STRING, GeneMANIA, cBioPortal, UALCAN, The Human Protein Atlas, Gene Expression Profiling Interactive Analysis (GEPIA), and Oncomine.

**Results:**

In CC tissues, CBX 2/3/4/5 and CBX 8 expression levels were considerably higher, whereas CBX 6/7 expression levels were lower. In CC, the CBX 5/6/8 promoters have elevated levels of methylation. The expression of CBX 2/6/8 and the pathological stage were connected. A 37% mutation rate of the differentially expressed CBX genes was observed. Also, there was a strong correlation of the CBXs expression with immune cell infiltration, such as T CD4^+^ cells, macrophages, neutrophils, B cells, T CD8^+^ cells, and dendritic cells.

**Conclusion:**

The investigation discovered that members of the CBXs family may be therapeutic targets for CC patients and may play significant roles in the development of CC tumors.

## Introduction

With more than 500,000 new cases and 260,000 fatalities each year, cervical cancer (CC) is considered the fourth most frequently diagnosed malignancy globally and the second major cause of mortality caused by tumors among women in developing nations (1, 2). According to epidemiological case studies, the human papillomavirus’s high-risk subtypes (HPV16 and HPV18) are virtually always the cause of CC (2). Additionally, CC development and tumorigenesis both need the activation of oncogenes and the inactivation of tumor suppressors. The primary reasons for treatment failure and mortality in CC patients are tumor invasion and distant metastases (3). The five-year survival rate of CC patients is 20-30% lower when pelvic lymph node metastases are present (4). Early diagnosis can considerably enhance the effectiveness of CC treatment. Thus, it is critical to identify more potent treatment targets and prognosis indicators for CC.

Through modifying the chromatin epigenetically and regulating a variety of physiological processes, such as transcriptional repression, apoptosis, cell cycle regulation, and developmental regulation, the Polycomb Repressive Complex (PRC) is typically made up of the Chromobox (CBX) family members (5). There are now eight proteins of the CBX family found in mammalian cells. Two categories of CBX family proteins are the polycomb (Pc) group, which includes CBX8, CBX7, CBX6, CBX4, and CBX2; and the heterochromatin protein 1 (HP1) group, which contains CBX1, CB3, and CBX5 (6). 

The proteins of the CBX family are implicated in tumor development and spread according to evidence (7). CBXs can have pro- or anti-tumor effects in different tumors and under different cellular environments. According to studies, CBX4 is up-regulated in breast cancer and activates the Notch1 signaling pathway to carry out carcinogenic actions (8). CBX4 inhibits the migration, invasion, and metastasis of colorectal cancer cells by luring Histone deacetylase 3 (HDAC3) to the promoter of RUNX family transcription factor 2 (Runx2) (9). CBX7 acts as a tumor suppressor gene in cervical, lung, colon, and thyroid cancer, but its function as an oncogene was also observed in gastric cancer (10). In cervical cancer cells, overexpression of CBX7 reduced cell migration and invasion caused apoptosis and suppressed cell proliferation (11). CBX7 provides reference value for the diagnosis and new targeted treatment of CC, while the detailed roles and the clinical values of CBXs in CC initiation and progression are totally unexplored.

Bioinformatics analysis has yet to be used to explore the association of CBXs in CC. Using online public databases, the infiltration of immune cells, changes in genes, possible roles, values of prognosis, clinicopathological characteristics, and the patterns of gene expression in CC patients were examined. We concluded that CBXs may have multiple and different activities in CC and may be used as the targets for the management and therapy of CC in clinics. 

## Materials and Methods

Since all the data were retrieved from the online databases, it could be affirmed that all written informed consent had already been obtained. Our study is based on open-source data, so there are no ethical issues or other conflicts of interest.

***Oncomine analysis***

To analyze the gene expression profiles in tumors and determine the sequences of DNA or RNA using a large microarray database of cancer, Oncomine (https://www.oncomine.org/resource/login.html) was applied (12). Using the Oncomine database, we assessed the expression of eight members of the CBX family in different types of cancer. The gene rank=10%, fold change >2, and *P*-value<0.05 were used to define the significant thresholds. The Student’s t-test was performed to compare the expression of CBXs in CC.

***Gene expression profiling interactive analysis (GEPIA)***

As a newly developed analytical tool, GEPIA (http://gepia.cancer-pku.cn/index.html) can analyze data from thousands of tumor and normal tissue samples using a standard processing pipeline (13). Herein, we analyzed the differentially expressed genes (DEGs) between normal and CC tissues and conducted the analyses of pathological stage and correlative prognosis via GEPIA. The *P*-value was calculated using the Student’s t-test.

***UALCAN***

As an intensive and extensive website, UALCAN (http://ualcan.path.uab.edu) can offer cancer data from The Cancer Genome Atlas (TCGA) database (14). To obtain the mRNA expression data, the methylation level of the promoter, and the connection of CBX expression to the clinicopathological parameters in patients with CC, the UALCAN was used. To define the differences, the *P*-value calculated by the Student’s t-test less than 0.05 was applied. 

***cBioPortal***

As a web resource that comprehensively provides visual and multidimensional cancer genomics data (15), cBioPortal (www.cbioportal.org) was conducted to obtain the network module of CBXs and genetic alterations using the TCGA database. 

***GeneMANIA***

As a resource-rich website, GeneMANIA (http://www.genemania.org) can prioritize genes for functional assays, analyze gene lists, and provide gene information using a prediction algorithm with high accuracy (16). We used it to weight indicators of CBX predictive value.

***STRING***

STRING (https://string-db.org/) is a protein interaction website that aims to create a comprehensive and objective global network and present users with a one-of-a-kind set of computational predictions (17). For the collection and integration of the potential interactions and different CBXs expressions, we constructed a PPI network using STRING. 

***TIMER***

For the evaluation of immune cell infiltrations and their impact in clinics in a systematic manner (18), TIMER (https://cistrome.shinyapps.io/timer/) was applied. CBXs were chosen for input via the “Gene module,” and then to show the association between CBX expression and the infiltration of immune cells in rectal and colon cancers, the scatterplots were generated. 

***Atlas of human proteins***

As a public database, the Human Protein Atlas (HPA) can provide information on the expression of mRNA and proteins in human healthy and malignant tissues, as well as blood cells and cell lines (19). Herein, using immunohistochemical imaging, we compared CBXs expression at the translational level between human CC and normal tissues directly. 

***Metascape***

Metascape (http://metascape.org) is an instrument to analyze the enrichment of gene lists and gene annotation (20). Using Metascape’s “Custom Analysis” module, we enriched CBX genes and related genes.

***Culture of cells***

CaSki, SiHa, and HeLa cell lines from the Chinese Academy of Sciences’ cell bank (Shanghai, China) were used to study human cervical cancer. The American Type Culture Collection (ATCC, USA) sold Ect1/E6E7. DMEM containing 10% FBS, 100 mg/ml streptomycin, and 100 U/ml penicillin was used to culture cells. All cells were cultured in a humidified incubator at 37 ^°^C containing 5% CO_2_.

***Extraction of RNA and qRT-PCR***

RNA was extracted using TRIzol reagent (Invitrogen). Then, qRT-PCR using the Real-Time SYBRTM Green PCR Master Mix kit (Applied Biosystems) was used to access gene expression. To standardize the levels of the target genes, GAPDH was employed as the housekeeping gene. Supplementary Table 1 presents primer sequences for qRT-PCR. Triplicates of each qRT-PCR experiment were performed. The 2^-ΔΔCt^ technique was used to examine the data. The means and standard deviation of the data were reported (SD). Utilizing SPSS, statistical analyses were performed. Student’s t-test was employed to compare groups. Statistics were deemed significant at *P*<0.05.

## Results

***Transcriptional levels of CBXs in CC***

**Figure 1:**
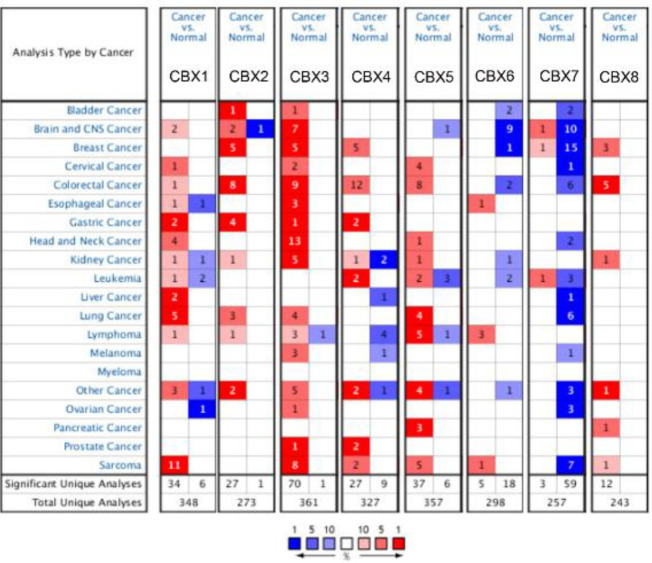
Transcriptional levels of the CBX family members in different cancers (Oncomine). The graphic demonstrates the numbers of datasets with statistically significant alterations in the mRNA expression of the target gene: up-regulated (red) and down-regulated (blue)

Using Oncomine, GEPIA, and UALCAN databases, we analyzed CBXs expression at the transcriptional level in normal and cancer tissues. In CC patients, CBX 7 was down-regulated, whereas the transcriptions of CBX 1, CBX 3, and CBX 5 were elevated in four datasets, through the Oncomine database (Figure 1). In the Pyeon dataset, CBX 1 had a fold change of 3.726 (*P*=1.72E-8); for CBX2 the fold change was 1.427 (*P*=0.004); for CBX 3, the fold change was 2.701 (*P*=2.37E-9); and CBX 5 was up-regulated with a fold change of 2.780 (*P*=1.29E-8). In the Zhai dataset, CBX 1 was overexpressed in High-Grade Cervical Squamous Intraepithelial Neoplasia Epithelia (HG-CSINE) with a fold change of 1.046 and in Cervical Squamous Cell Carcinoma (CSCC) Epithelia with a fold change of 1.337 (*P*=5.62E-4); CBX 3 was overexpressed in HG-CSINE (fold change=1.576 and *P*=0.003) and in CSCC Epithelia (fold change=1.911 and *P*=1.26E-8); for CBX 5, it was overexpressed in HG-CSINE (fold change=2.166 and *P*=5.28E-4) and in CSCC Epithelia (fold change=2.180 and *P*=5.45E-4). According to the Scotto dataset, CBX 1 was increased in CSCC with a fold change of 1.364 (*P*=0.003); CBX4 was up-regulated in CSCC (fold change=1.217 and *P*=0.002); and CBX 5 was found to be up-regulated in CSCC (fold change=2.094 and *P*=4.15E-6). In the Biewenga dataset, CBX 1 was overexpressed in invasive breast cancer compared with normal tissue with a fold change of 1.159 (*P*=0.018); CBX 3 was increased in CSCC with a fold change of 2.202 (*P*=1.13E-5), and CBX 5 was also increased in CSCC (fold change=1.318 and *P*=0.004).

In three datasets, CC patients had increased CBX 4 mRNA expression: in the Zhai dataset CBX 4 was found to be overexpressed in HG-CSINE (fold change=1.242 and *P*=0.046); in the Pyeon dataset CBX 4 was found to be higher in CC (fold change=1.324, *P*=0.021). In two datasets, CBX 6 mRNA expression was higher in CC patients: it was found to be overexpressed in HG-CSINE (fold change=1.613, *P*=2.13E-4), as well as CSCC Epithelia (fold change=1.333, *P*=0.049); in Biewenga dataset CBX 6 was found to be up-regulated in CSCC (fold change=1.43 and *P*=0.012). In one dataset, CBX 8 mRNA expression was higher in CC patients. Table 1 summarizes the findings.

**Figure 2:**
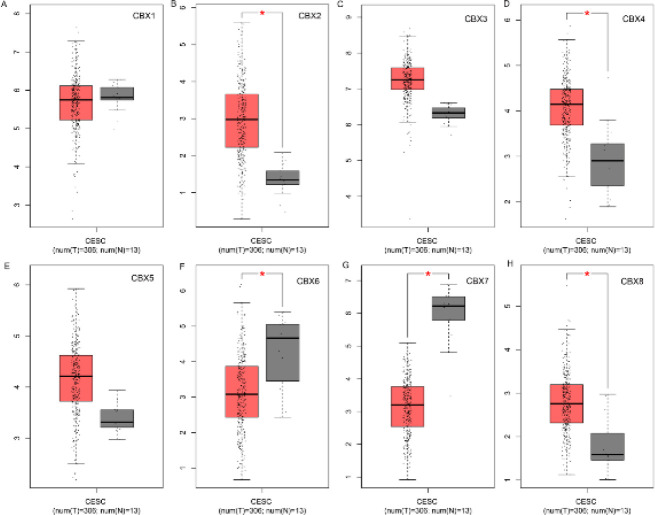
Expression of CBX family members in cervical cancer (GEPIA), *P< 0.05

The transcript levels of eight members of the CBX family were then assessed in the GEPIA database. In CC tissues, CBX 2, CBX 4, and CBX 8 expression was considerably up-regulated (*P*=0.05) compared with normal samples, but mRNA expression of CBX 6 and CBX 7 was significantly down-regulated (*P*=0.05) (Figure 2).

**Figure 3:**
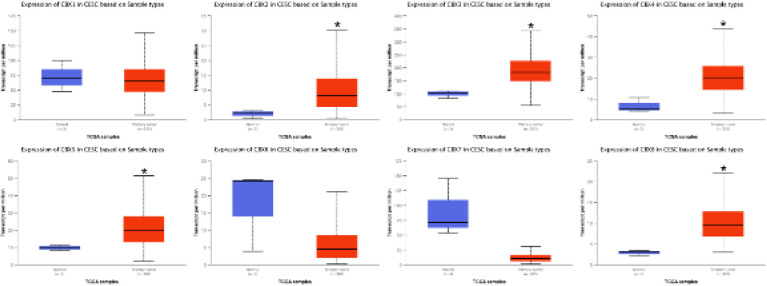
Transcription levels of CBX family members in cervical cancer (UALCAN)

Furthermore, by using the UALCAN database, in comparison with the normal tissues, the CC tissues exhibited dramatically elevated expression of CBX 8, CBX 5, CBX 4, CBX 3, and CBX 2 (Figure 3). Additionally, among all the studied CBXs, CBX 3 showed the highest expression in CC tissues (Supplementary Figure 1).

**Figure 4:**
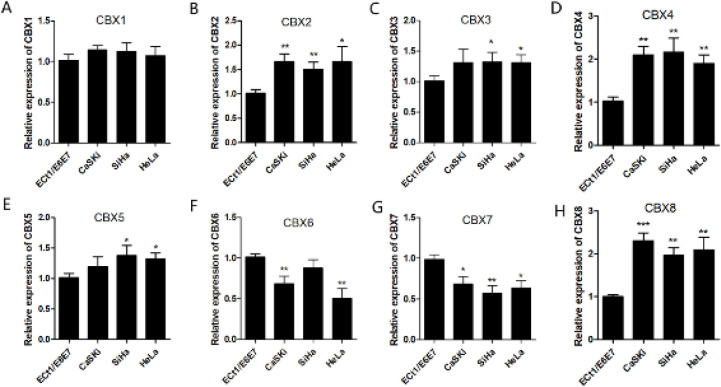
mRNA expressions of eight CBX family members in human CC cell lines. The mRNA expressions of eight CBX family members (CBX1/2/3/4/5/6/7/8) in human CC cell lines (CaSKi, SiHa, and HeLa) and human cervical immortalized squamous cell line (ECt1/E6E7) by qRT-PCR assays. ECt1/E6E7 was used as a control

Finally, qRT-PCR assays were used to detect the mRNA expression of eight CBX family members (CBX 1/2/3/4/5/6/7/8) in human CC cell lines (CaSKi, SiHa, and HeLa) and human cervical immortalized squamous cell line (ECt1/E6E7). We observed dramatically increased transcription of CBX 8, CBX 5, CBX 4, CBX 3, and CBX 2 in CC cells (Figure 4). 

***Levels of CBX promoter methylation in CC***

We investigated the methylation levels of CBX promoters in CC using the UALCAN database and found that CBX 8, CBX 6, and CBX 5 promoters were hypermethylated in CC (Supplementary Figure 2).

***CBX protein expression in CC***

**Figure 5:**
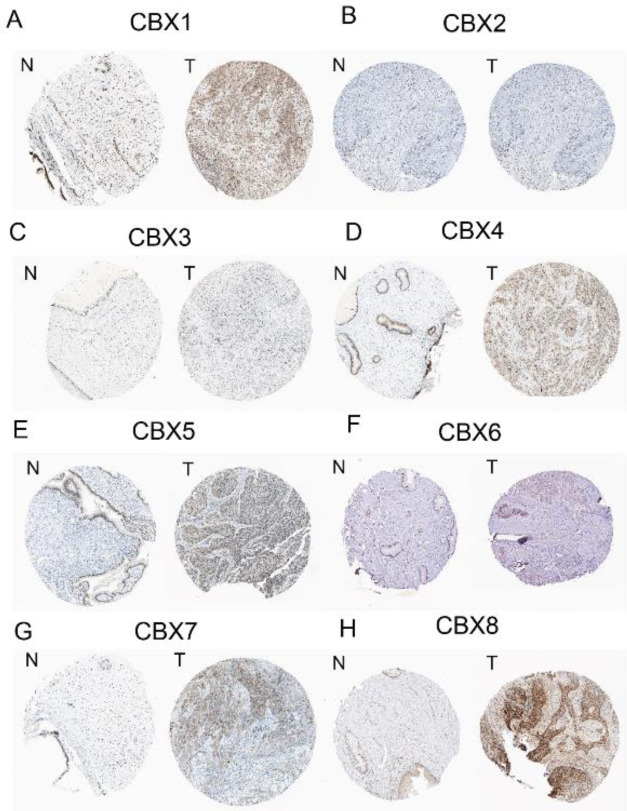
Protein expression of CBX family members in normal cervical tissues and cervical cancer tissues (Human Protein Atlas)

Using the HPA, we observed significantly elevated expression of CBX 1, CBX 4, CBX 5, CBX 7, and CBX 8 in CC tissues in comparison with those of normal tissues at the translational level (Figure 5). However, no significant differences in CBX 2, CBX 3, and CBX 6 expressions were found among normal and CC tissues. 

***Relationship of the mRNA levels of CBX with the clinicopathological parameters of CC patients***

**Figure 6:**
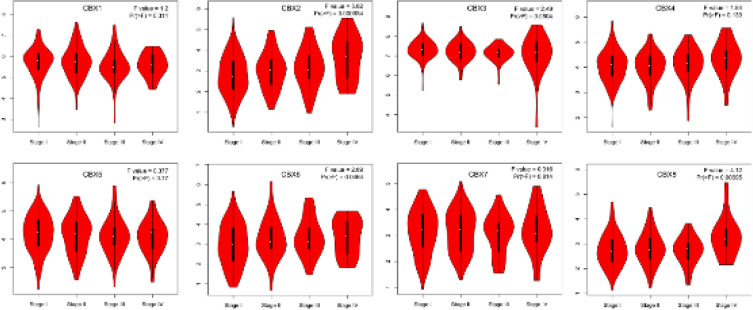
Correlations between CBX expression and tumor stage in cervical cancer patients (GEPIA)

After determining the expression of each CBX family member in CC, we examined the relationship of the mRNA levels of CBX with the pathological stage of CC patients and discovered a close connection of the expression of CBX 2 (*P*=0.000934), CBX 6 (*P*=0.0464), and CBX 8 (*P*=0.00695) to the pathological stage (Figure 6), where the expression levels increased as the tumor progressed. These observations suggested that CBX family members play an important role in CC tumorigenesis and progression. 

Regarding that, the UALCAN database was used to examine the connections of the mRNA levels of CBX with the clinicopathological parameters of CC patients, such as individual cancer stages, tumor grade, and nodal metastasis status. CBX mRNA expression was found to be significantly related to the cancer stage. Patients with advanced cancer stages tended to have higher CBX 2, CBX 3, CBX 4, CBX 5, and CBX 8 expressions, and lower CBX 6 expression (Supplementary Figure 3). The significant relations of CBX 4, CBX 5, and CBX 8 expressions with the status of nodal metastasis were also observed (Supplementary Figure 4). Furthermore, significant relation between CBX 1 and CBX 6 expression with the tumor grade was also observed in CC patients (Supplementary Figure 5).

***The value of CBX expression for the prognosis of CC Patients***

**Figure 7:**
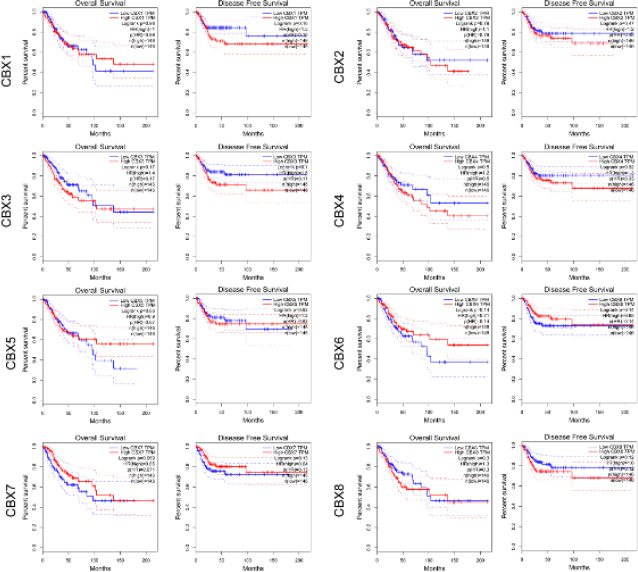
Prognostic value of the mRNA expression of distinct CBX family members in CC (GEPIA)

To assess the value of differentially expressed CBX in the progression of CC, we analyzed the connections of CBX to the clinical outcomes by using GEPIA. As shown in Figure 7, no significant connections of these CBX expressions to the overall survival (OS) and disease-free survival (DFS) were found. 

**Figure 8:**
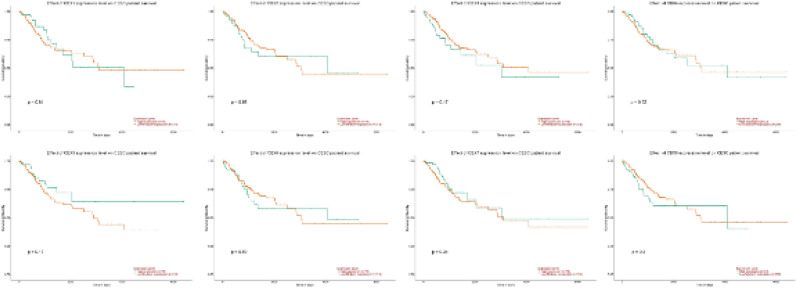
Prognostic value of the mRNA expression of distinct CBX family members in CC (UALCAN)

In addition, we used UALCAN to assess the prognostic value of eight CBX in patients with CC (Figure 8). Similarly, no connections of these CBX to the OS were found in CC patients.

***Genetic alteration, expression, and interaction analyses of CBXs in CC patients***

**Figure 9:**
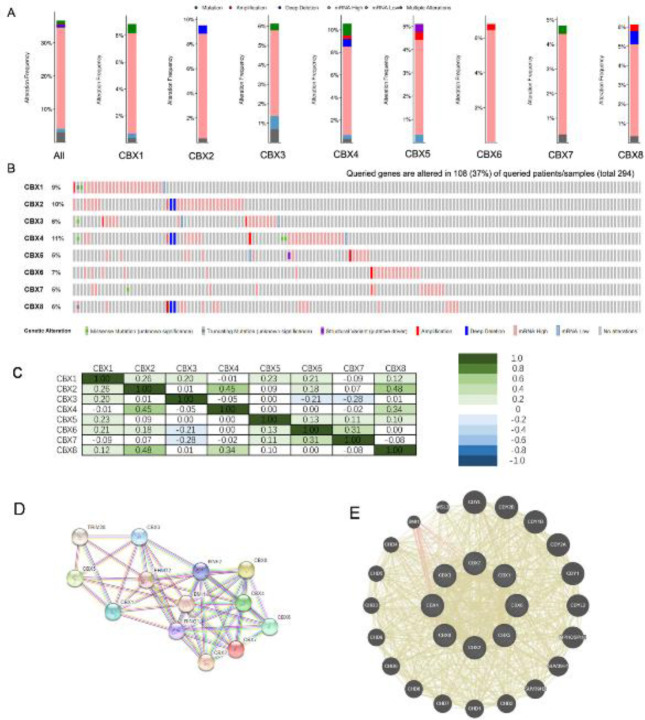
Genetic alteration, neighbor gene network, and interaction analyses of different expressed CBX family members in CC patients. (A, B) Summary of alterations in different expressed CBX family members in CC (cBioPortal). (C) Correlation heat map of different expressed CBX family members in CC. (D, E) Protein-protein interaction network of different expressed CBX family members (GeneMANIA, STRING)

We thoroughly examined the molecular properties of the differentially expressed CBX family members. The cBioPortal online tool was used to examine the genetic changes in CBX in CC patients. As shown in Figure 9, we obviously observed many changes (no less than 2) in several subtypes of CC, moreover, we also found more prevalent depletion changes in the samples from CC (Figure 9A). With a mutation incidence of 37%, 108 out of the 294 sequenced CC patients had genetic changes. The OncoPrints contained high and low amounts of mRNA levels as well as amplification, deletion, fusion, truncating mutation, and missense mutation. As shown in Figure 9B, the genetic change rates of these CBXs, such as CBX 8, CBX 7, CBX 6, CBX 5, CBX 4, CBX 3, CBX 2, and CBX 1, were 6%, 5%, 7%, 5%, 11%, 6%, 10%, and 9%, respectively (Figure 9B). Additionally, in these samples, the frequently observed changes were elevated gene expression. 

Still, by using cBioPortal we also determined the correlations between CBXs by assessing their mRNA expressions (RNA sequencing [RNA-seq] version 2 RSEM) and applying Pearson’s correction. The findings showed that the following CBXs had substantial and positive correlations: CBX 4 with CBX 2, CBX 8 and CBX 2, CBX 8 with CBX 4, and CBX 7 and CBX 6 (Figure 9C).

Additionally, to investigate potential connections between the eight CBXs family proteins that were differentially expressed, we used the STRING database to conduct a protein-protein interaction (PPI) network analysis (Figure 9D). The GeneMANIA results also showed that the main effects of these CBXs and CBXs-related proteins, such as CDYL, CDY2B, CDY1B, CDY2A, CDY1, CDYL2, MPHOSPH8, SUV39H1, SUV39H2, CDH1/2/3/4/5/6/7/8/9, BMI1, and MSL3, are PRC1 complex, heterochromatin, nuclear chromosome, and histone binding (Figure 9E).

***Functional enrichment analysis of CBXs in CC patients***

**Figure 10:**
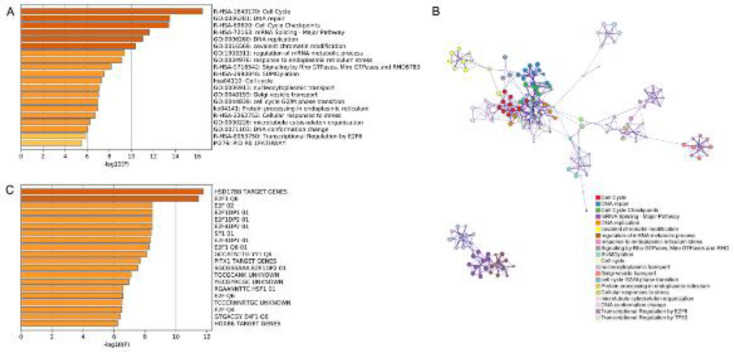
Predicted functions and network of enriched terms of CBX genes and similar genes. (A) Gene Ontology (GO) analysis. (B) Network of enriched terms of CBX genes and similar genes. (C) Summary of enrichment analysis in Transcription Factor Targets

To identify the CBX-like genes in the tissues of CC patients, the GEPIA was employed first. For each CBX gene, we used the first 100 similar genes. We then used Metascape to conduct gene ontology (GO), KEGG, and PPI enrichment analyses. The pathways and functions of these CBXs and CBXs-related genes prediction are depicted in Figures 10A and B. These genes were found to be involved in a variety of molecular functions, cellular components, and biological processes. The following GO terms were found to be significantly enriched in the genes related to cell cycle, DNA repair, cell cycle checkpoints, mRNA splicing-major pathway, DNA replication, and covalent chromatin modification. Figure 10C depicts the summary of enrichment analysis in transcription factor targets.

***Connection of immune cell infiltration to CBXs in patients with CC***

**Figure 11:**
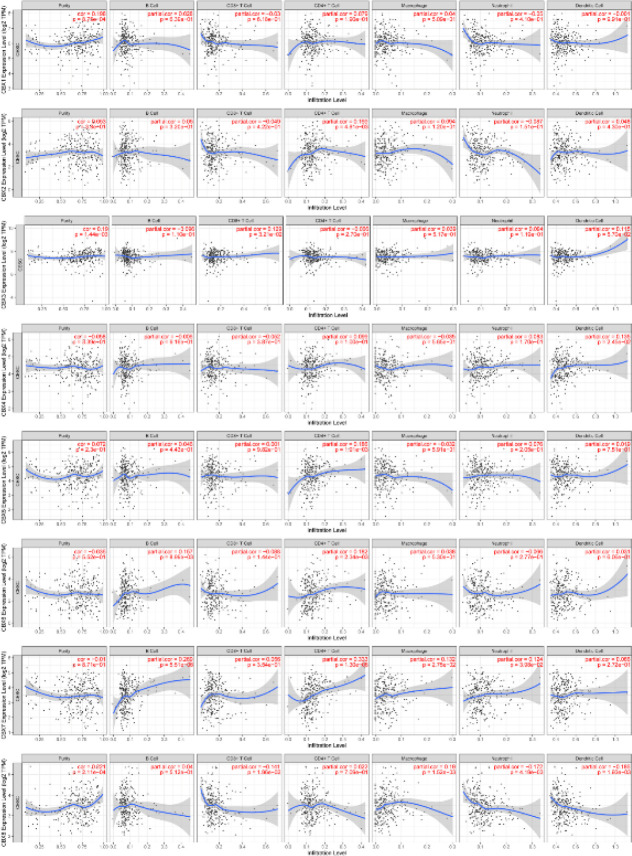
Relationship between differentially expressed CBX family members and immune cell infiltration. The immune cells we analyzed included B cells, CD8+T cells, CD4+T cells, macrophages, neutrophils, and dendritic cells

The clinical outcomes of cancer patients are significantly affected by the infiltration of immune cells and inflammatory responses. Using the TIMER database, we examined the relationship between eight CBXs expression levels and immune cell infiltration. CBX 1, CBX 2, and CBX 6 expressions were positively associated with B cell, T CD4^+^ cell, macrophage, and dendritic cell infiltration and negatively associated with T CD8^+^ cell and neutrophil infiltration, as shown in Figure 11. CBX 3 expression was found to be negatively associated with B cell and T CD4^+^ cell infiltration and positively associated with dendritic cell, neutrophil, macrophage, and T CD8^+^ cell infiltration. CBX 4 expression was associated with the infiltration of macrophage, T CD8^+^ cell, and B cell, but not with T CD4^+^ cell, neutrophil, or dendritic cell infiltration. Except for macrophages, the infiltrations of dendritic cells, neutrophils, T CD4^+^ cells, T CD8^+^ cells, and B cells were significantly associated with CBX 5 expression. Additionally, the infiltrations of dendritic cells, neutrophils, macrophages, T CD4^+^ cells, T CD8^+^ cells, and B cells were also correlated with CBX 7 expression. There was a negative correlation between CBX 8 expression and the infiltration of T CD8^+^ cells, neutrophils, and dendritic cells, and a positive correlation between CBX 8 expression and infiltration of B cells, T CD4^+^ cells, and macrophages. Moreover, we also observed a significant connection of CBX 3 to the clinical outcome of the patients with CC using the Cox proportional hazard model after adjustment with confounding factors (Table 2). 

## Discussion

CBX family proteins, as important components of epigenetic regulation complexes, can play a role in the emergence and progression of various tumors. CBXs can exhibit a promotive or suppressive effect in different types of tumors and under different cellular conditions. 

We investigated eight CBXs expressions and their relationship to the pathological stage in CC and discovered dramatically elevated expression of CBX 8/5/4/3/2/1 and reduced expression of CBX 7/6 in CC tissues. Furthermore, in CC, the hypermethylation of CBX 8/6/5 promoters was also observed. We also looked at the relationship of the differentially expressed CBXs with the pathological stage of CC patients and discovered a significant link between CBX 2, CBX 6, and CBX 8 expression and the pathological stage. CBX 2, CBX 6, and CBX 8 expression increased as the tumor progressed. Due to the significant differential expression in CC patients, the molecular characteristics of these CBXs were investigated in CC tissue. A frequent genetic alteration (mutation rate=37%) of these CBXs was observed. The most significant change was increased mRNA expression. The pathogenesis and development of CC are complex and multifaceted processes, with genetic changes playing an important role. A growing body of evidence suggests that the tumor microenvironment (TME) can influence the occurrence and progression of tumors. The suppressive or promotive effect of the immune cells in TME was also observed. We examined the relationship between eight CBXs expression levels and immune cell infiltration in this study and discovered that CBX expression in CC was significantly correlated with dendritic cells, T CD8^+^ cells, neutrophils, B cells, T CD4^+^ cells, and macrophages.

According to previous research, CBX 1 expression was elevated in HCC tissues and was associated with poor outcomes. Through the activation of the Wnt/β-Catenin pathway and the interaction with HMGA2, the migration and proliferation of HCC cells were significantly enhanced by CBX 1 overexpression (21). CBX 1 appears to be a potential prognostic biomarker for HCC as well as an oncogene that promotes HCC progression, according to these findings. According to other research, CBX 2 is a novel cancer biomarker (22-24). CBX 2 expression increased progressively through the mucosa-adenoma-carcinoma sequence, suggesting that it could be used as a diagnostic biomarker and therapeutic target for CRA and CRC (23). In breast cancer, increased CBX 2 expression was substantially correlated with positive HER-2 status, high tumor, node, metastasis (TNM) stage, lymph node metastases, and bigger tumor size (24). 

According to our findings, to determine the prognosis and clinicopathological characteristics of patients with a variety of human malignancies, CBX 3 expression can be employed as a brand-new biomarker. In a meta-analysis of eleven studies involving 1682 cancer patients, higher CBX 3 expression was substantially linked to a shorter OS (25). The elevation of CBX 3 expression was linked to poor OS in stomach cancer, lung cancer, genitourinary cancer, and tongue squamous cell carcinoma. Additionally, elevated CBX 3 expression was also linked to lymph node metastases and larger tumor size in terms of clinicopathological characteristics. Through its interaction with HDAC1, CBX 4 causes the tumor suppressor KLF6 to be transcriptionally repressed in ccRCC (26). The Wnt/β-catenin pathway also performs an oncogenic role for CBX 4, which makes it a possible therapeutic target for lung cancer (27). Additionally, CBX 4 is expressed more frequently in breast cancer and uses the Notch1 signaling pathway to carry out carcinogenic functions (8). Targeting the CBX 4/miR-137 axis may be therapeutically effective in the treatment of breast cancer because CBX 4 serves as a predictive biomarker. Patients with high CBX 4 expression should receive postoperative TACE treatment to increase their chance of survival because CBX 4 is a standalone prognostic factor for HCC patients (28). 

MiR-589-targeting 5p’s of CBX 5 may promote cell migration and proliferation in Renal Cell Carcinoma (RCC) (29). The inhibitory effects of suppressed LOXL1-AS1 on RCC malignant traits were reversed when CBX 5 was overexpressed or when miR-589-5p was repressed. When compared with non-metastatic breast cancer, CBX 5 is transcriptionally and proteinically down-regulated in metastatic breast cancer. Previous research indicates that CBX 6 can suppress the progression of breast cancer (30). Breast cancer cells’ migration and invasion were suppressed, and exogenous overexpression of CBX 6 reduced colony formation, cell proliferation, and cell cycle arrest *in vitro* (31). However, CBX 6 expression was found to be substantially linked to greater tumor sizes and numerous tumors in clinical samples and cell lines of HCC (32). Both *in vitro *and *in vivo* studies also indicated that CBX 6 profoundly participated in the promotion of HCC growth. 

Clinically, CBX 7 expression varies for cancer progression in various malignancies and exhibits double-sidedness. Malignancies of the breast, pancreas, liver, thyroid, colon, and glioma express CBX 7 at modest levels, but cancers of the stomach, prostate, and lymphoid tissues express it at high levels (10). According to the various tissue contexts, CBX 7 engages in several pathways for its anti-cancer impact. It mainly interacts with other molecules to regulate some cell signaling pathways to prevent cancer symptoms by epigenetically controlling the expression of associated proteins. CBX 7 may act as a tumor suppressor and represent a possible target in cervical cancer (33). According to our findings, CC tissues also had considerably lower amounts of CBX 7 mRNA and protein. According to a previous study, CBX 8 is significantly linked with the FIGO stage and is highly expressed in cervical cancer. In comparison with the patients with low CBX 8 expression, the high CBX 8 expressed patients exhibited dramatically decreased DFS and OS (34). CBX 8 may therefore be a standalone predictive factor for cervical cancer patients. The amounts of CBX 8 mRNA and protein in normal tissues and cervical cancer tissues were compared in our investigation, but we were unable to detect any significant differences.

Our current study could provide detailed immunization data to aid in the development of new immunotherapies. Using GO enrichment analysis, we finally focused on the function of these CBXs. These genes’ functions are primarily related to the Cell Cycle, DNA repair, Cell Cycle Checkpoints, mRNA Splicing-Major Pathway, DNA replication, and covalent chromatin modification, according to the findings. According to the findings, these CBXs family members significantly participated in the pathogenesis and progression of CC. 

Some limitations of this study should be considered when interpreting the current results. Because the data analyzed by us were obtained from online databases, additional clinical and experimental studies should be done to verify our conclusions. Moreover, the underlying mechanisms, clinical applications, and interactions between molecules of these CBXs in CC should also be clarified. 

To the best of our knowledge, this is the first study to systematically examine their expression at mRNA and protein levels, as well as their associations with potential functions, genetic alterations, prognostic values, and clinicopathological parameters in CC. We believe that our research will help improve CC patients’ early diagnosis, treatment outcomes, and prognosis. 

**Table 1:** The mRNA levels of CBXs in different types of cervical cancer tissues and normal cervical tissues at transcriptome level

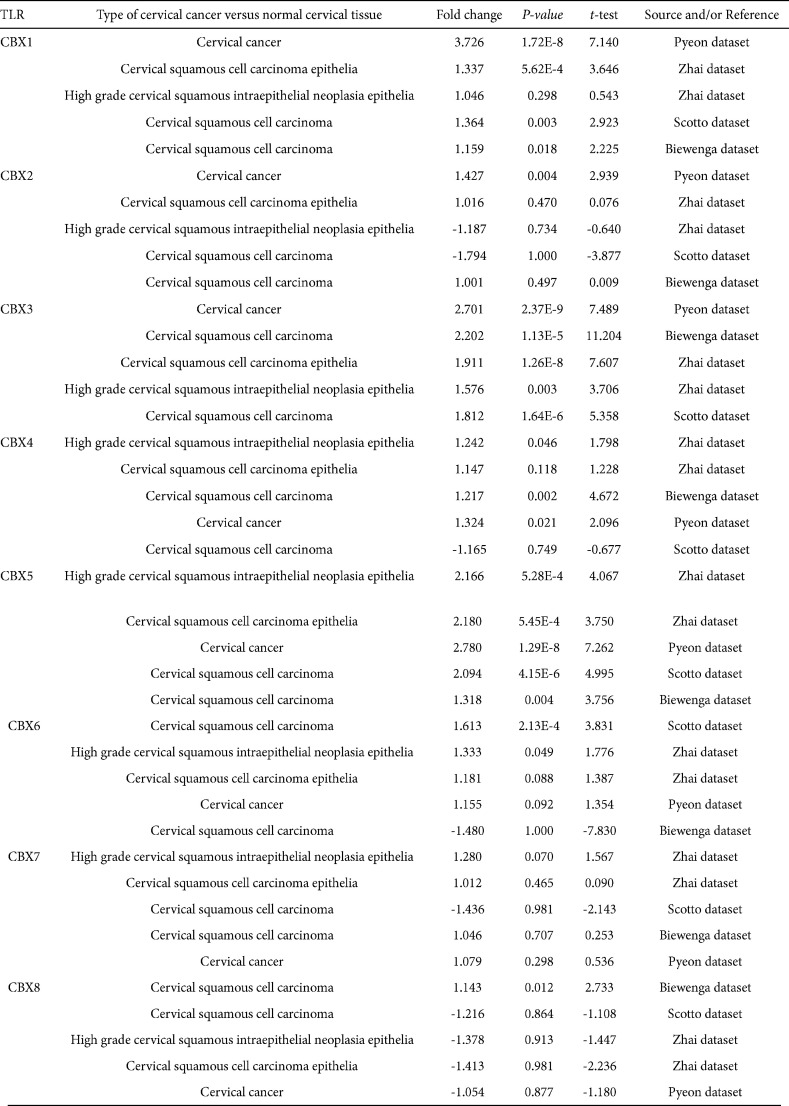

**Table 2:** The Cox proportional hazard model of CBXs chemokines and six tumor-infiltrating immune cells in cervical cancer (TIMER)

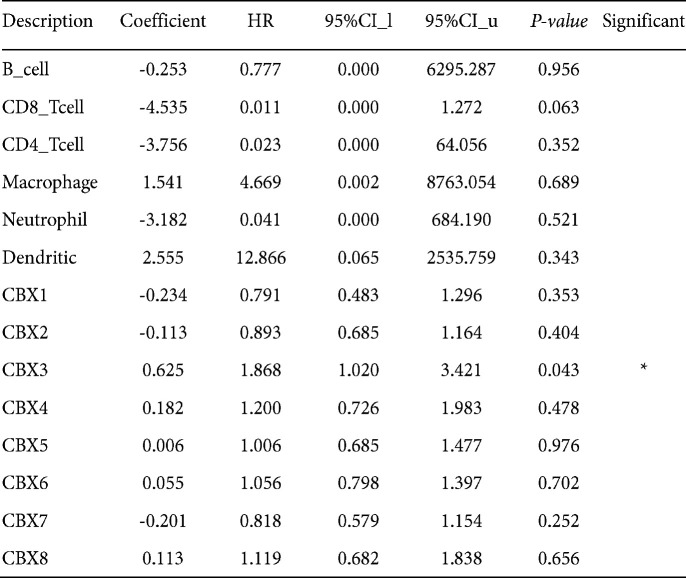

* *P<*0.05

## Conclusion

In the present study, we focused on the expression change and the prognostic value of CBXs, as well as their connections to the infiltration of immune cells in CC. We also discovered significantly elevated CBX 8/5/4/3/2/1 expression and reduced CBX 7/6 levels in CC tissues. CBX 5, CBX 6, and CBX 8 promoters were hypermethylated in CC. CBX 2, CBX 6, and CBX 8 expressions were linked to the pathological stage. Additionally, significant associations of CBXs expression with the infiltration of various immune cells, such as dendritic cells, T CD8^+^ cells, B cells, neutrophils, macrophages, and T CD4^+^ cells in CC were also found. Our findings suggest that the CBX family may play important roles in CC tumorigenesis and may be therapeutic targets for CC patients.

## Authors’ Contributions

Dl participated in the design of this study, carried out the experiments, performed the statistical analysis, and drafted the manuscript. XL, LH, YY, XY, and PF performed the experiments and analyzed the data. CL and YL analyzed and interpreted data, and supervised, directed, and submitted the paper. All authors have read and approved the final version of the manuscript.

## Conflicts of Interest

The authors have no conflicts of interest to declare. 
